# The WRN exonuclease domain protects nascent strands from pathological MRE11/EXO1-dependent degradation

**DOI:** 10.1093/nar/gkv836

**Published:** 2015-08-14

**Authors:** Chiara Iannascoli, Valentina Palermo, Ivana Murfuni, Annapaola Franchitto, Pietro Pichierri

**Affiliations:** 1 Section of Experimental and Computational Carcinogenesis, Department of Environment and Primary Prevention, Istituto Superiore di Sanità, Viale Regina Elena 299, 00161 Rome, Italy; 2 Section of Molecular Epidemiology, Department of Environment and Primary Prevention, Istituto Superiore di Sanità, Viale Regina Elena 299, 00161 Rome, Italy

## Abstract

The WRN helicase/exonuclease protein is required for proper replication fork recovery and maintenance of genome stability. However, whether the different catalytic activities of WRN cooperate to recover replication forks *in vivo* is unknown. Here, we show that, in response to replication perturbation induced by low doses of the TOP1 inhibitor camptothecin, loss of the WRN exonuclease resulted in enhanced degradation and ssDNA formation at nascent strands by the combined action of MRE11 and EXO1, as opposed to the limited processing of nascent strands performed by DNA2 in wild-type cells. Nascent strand degradation by MRE11/EXO1 took place downstream of RAD51 and affected the ability to resume replication, which correlated with slow replication rates in WRN exonuclease-deficient cells. In contrast, loss of the WRN helicase reduced exonucleolytic processing at nascent strands and led to severe genome instability. Our findings identify a novel role of the WRN exonuclease at perturbed forks, thus providing the first *in vivo* evidence for a distinct action of the two WRN enzymatic activities upon fork stalling and providing insights into the pathological mechanisms underlying the processing of perturbed forks.

## INTRODUCTION

Replication fork perturbation or stalling commonly occurs during the duplication of complex genomes. Inaccurate handling of perturbed replication forks can result in fork inactivation, DNA double-strand break (DSB) generation and genome instability (1). Studies in model organisms, and most recently in human cells, indicated that stalled replication forks can be recovered through multiple mechanisms, most of which require processing of the forked DNA by helicases, translocases or nucleases (2–4). Furthermore, recombination plays a crucial role in the recovery of stalled forks either through their stabilization or by promoting repair of DSBs induced when stalled forks collapse (5). Although many of the components of these pathways have been identified, little is known about the molecular mechanisms underlying replication fork recovery under normal or pathological conditions. One of the events occurring at stalled forks, which was first identified in bacteria, is the regression of the stalled replication fork to form a four-way structure characterized by pairing of the two extruded nascent strands (6). Such a reversed fork is a versatile structure that can be further processed by helicases or nucleases to restore a functional replication fork or be used by recombination enzymes for the recovery of replication (6). Biochemical experiments, and, most recently, electron microscopy of replication intermediates prepared from cultured cells contributed to the identification of some proteins involved in replication fork reversal in humans (7). In particular, recent studies demonstrated that regressed forks are easily formed upon treatment of cells with nanomolar doses of camptothecin (CPT), and that they are stabilized and recovered through a mechanism involving PARP1 and the RECQ1 helicase (8,9). However, the fate of a reversed fork under pathological conditions, that is when some of the enzymatic activities involved in its restoration are absent or the corresponding genes are mutated, is unclear. Seminal studies in recombination or checkpoint-defective yeast strains have evidenced that regressed forks undergo degradation by EXO1 and/or DNA2 (10,11). Degradation at stalled forks has also been reported in human cells with mutation in or depletion of BRCA2, RAD51 or FANCD2, but such extensive degradation would involve the MRE11 exonuclease (12,13). Interestingly, RAD51 could both prevent pathological degradation by MRE11 and stimulate the physiological processing of reversed forks by DNA2 (14,15), suggesting that MRE11 does not act on regressed forks, at least in the absence of RAD51.

It is not known whether MRE11-dependent degradation at perturbed forks is restricted to loss of the BRCA2/RAD51/FANC axis or is a general pathological response to impaired recovery of stalled forks; it is also unclear whether EXO1 or DNA2 is involved in this process.

The Werner syndrome helicase/exonuclease, WRN, is one of the proteins that is crucial for replication fork recovery *in vivo*, and that has shown the ability to regress and restore a replication fork *in vitro* (16–18). While coordinated action of both WRN catalytic activities could be involved in processing of replication fork regression *in vitro*, the helicase may be relevant for fork reversal, with the exonuclease being essential only for enlarging the template ssDNA region or to process the reversed fork (19–21). It is still unclear whether WRN functions at perturbed forks both to promote correct recovery from replication arrest and to prevent pathological degradation of nascent DNA.

Here, we explored the role of the WRN enzymatic activities in replication fork progression, stability and recovery in response to nanomolar concentrations of CPT and in the absence of DSBs. We found that the two enzymatic activities of WRN play distinct roles in protecting CPT-perturbed forks. The WRN exonuclease prevents unscheduled degradation of the nascent strands by the combined action of the MRE11 and EXO1 exonucleases, thus supporting the restart of stalled forks and limiting chromosome breakage.

In conclusion, we describe a previously undisclosed function of WRN exonuclease activity in counteracting unscheduled degradation of nascent strands, likely at regressed forks, thus contributing to the mechanistic appreciation of how incorrect processing of perturbed forks may correlate with genome instability.

## MATERIALS AND METHODS

### Cell lines and culture conditions

The SV40-transformed WRN-deficient fibroblast cell line (AG11395) was obtained from Coriell Cell Repositories (Camden, NJ, USA). AG11395 (WS) fibroblasts that were retrovirally transduced with full-length cDNA encoding wild-type WRN (WRN-WT), the missense-mutant form of WRN with inactive exonuclease (WRN-E84A) or helicase (WRN-K577M) were generated and characterized in a previous work (22). The SV40-transformed MRC5 fibroblast cell line (MRC5SV40) was a generous gift from Dr P. Kannouche (IGR, Villejuif, France).

All the cell lines were maintained in Dulbecco's modified Eagle's medium (DMEM; Life Technologies) supplemented with 10% FBS (Boehringer Mannheim) and were incubated at 37°C in a humidified 5% CO_2_ atmosphere.

### Chemicals

Camptothecin (Sigma-Aldrich) was dissolved in DMSO and a stock solution (10 mM) was prepared and stored at −20°C. Iododeoxyuridine (IdU) and chlorodeoxyuridine (CldU) (Sigma-Aldrich) were dissolved in sterile DMEM to obtain stock solutions of 2.5 mM and 200 mM, respectively, and stored at −20°C. Mirin (Calbiochem), an inhibitor of MRE11 exonuclease activity, was used at 50 μM; the B02 compound (Selleck), an inhibitor of RAD51 activity, was used at 27 mM. The CDK inhibitor roscovitine (Selleck) was used at 20 μM.

### DNA fibre analysis

Cells were pulse-labelled with 25 μM CldU and then labelled with 250 μM IdU with or without treatment, as shown in the experimental schemes. DNA fibres were prepared and spread out as previously described (18). For immunodetection of labelled tracts, the following primary antibodies were used: rat anti-CldU/BrdU (Abcam) and mouse anti-IdU/BrdU (Becton Dickinson). Images were acquired randomly from fields with untangled fibres using the Eclipse 80i Nikon Fluorescence Microscope, equipped with a VideoConfocal (ViCo) system. The lengths of labelled tracts were measured using the Image-Pro-Plus 6.0 software. A minimum of 100 individual fibres were analysed for each experiment and each experiment was repeated three times. In dot plots, the mean of at least three independent experiments are presented. The value of the IdU tract length is reported in micrometers.

### Neutral comet assay

The neutral comet assay was performed as previously described (23). Cell DNA was stained with GelRed (Biotium) and examined at 40× magnification with an Olympus fluorescence microscope. Slides were analysed with a computerized image analysis system (Comet IV, Perceptive UK). To assess the amount of DNA DSB breaks, computer-generated tail moment values (tail length × fraction of total DNA in the tail) were used. A minimum of 200 cells was analysed for each experimental point. Apoptotic cells (smaller comet head and extremely larger comet tail) were excluded from the analysis to avoid artificial enhancement of the tail moment.

### Detection of nascent single-stranded DNA

To detect nascent single-stranded DNA (ssDNA), cells were labelled for 30 min with 250 μM IdU (Sigma-Aldrich), immediately prior to the end of the CPT treatments. For immunofluorescence, cells were washed with PBS, permeabilized with 0.5% Triton X-100 for 10 min at 4°C, and fixed as previously described (22). Fixed cells were then incubated with mouse anti-IdU antibody (Becton Dickinson) for 1 h at 37°C in 1% BSA/PBS, followed by incubation with species-specific fluorescein-conjugated secondary antibodies (Alexa Fluor 488 Goat Anti-Mouse IgG (H+L), highly cross-adsorbed; Life Technologies). Slides were analysed with an Eclipse 80i Nikon Fluorescence Microscope, equipped with a VideoConfocal (ViCo) system. For each time point, at least 100 nuclei were examined by two independent investigators and foci were scored at 60×. Quantification was carried out using the ImageJ software. Only nuclei showing more than 10 bright foci were counted as positive. The results for parallel samples incubated either with the appropriate normal serum or only with the secondary antibody confirmed that the fluorescence pattern observed was not attributable to artefacts.

### RNA interference and chemical inhibition

RNAi oligos against EXO1 (Cat. N. SI02665138—EXO1#1 and Cat. N. SI02665145—EXO1#2), RECQ1 (Cat. N. GS5965, SMARCAL1 (Cat. N. GS50485—equimolar mixture of 4 the siRNAs) and MRE11 (Cat. N. SI02665180) were from Qiagen. Oligos were used at the final concentration of 40, 10, 20 and 20 nM, respectively. The siRNA against DNA2 was a kind gift from Dr Vindigni (14). Transfection was performed using INTERFERin (Polyplus) according to the manufacturer's instructions. As a control, a siRNA duplex directed against GFP was used. DNA2 was silenced using the pLKO-shDNA2 plasmid (Addgene #31951). The plasmid was transfected by nucleofection using the Neon system (Life Technologies). Decrease in protein levels was confirmed by western blotting at 48 h after transfection.

### *In situ* proximity ligation assay

The *in situ* proximity ligation assay (PLA) in combination with immunofluorescence microscopy was performed using the Duolink II Detection Kit with anti-Mouse PLUS and anti-Rabbit MINUS PLA Probes, according to the manufacturer's instructions (Sigma-Aldrich) (24). To detect proteins we used rabbit anti-WRN (Abcam) and rabbit anti-MRE11 (Novus Biological) antibodies. IdU-substituted ssDNA was detected with the mouse anti-BrdU antibody (Becton Dickinson) used in the DNA fibre assay.

### Immunoprecipitation and western blot analysis

Immunoprecipitation experiments were performed as previously described (25). Lysates were prepared from 2.5 × 10^6^ cells using RIPA buffer (0.1% SDS, 0.5% Na-deoxycholate, 1% NP40, 150 mM NaCl, 1 mM EDTA, 50 mM Tris/Cl, pH 8) supplemented with phosphatase, protease inhibitors and benzonase. One milligram of lysate was incubated overnight at 4°C with BcMag^TM^ Magnetic Beads (Bioclone) conjugated with 4 μg of anti-RECQ1 antibody under rotation, according to the manufacturer instructions. After extensive washing in RIPA buffer, proteins were eluted in 2× electrophoresis buffer and subjected to SDS–PAGE and western blotting.

Western blotting were performed using standard methods. Blots were incubated with primary antibodies against RECQ1 (Santa Cruz Biotechnology), SMARCAL1 (Bethyl), MRE11 (Novus Biological), DNA2 (Abcam), EXO1 (Santa Cruz Biotechnology), anti-PAR (Abcam), tubulin (Sigma-Aldrich) and lamin B1 (Abcam). After incubations with horseradish peroxidase-linked secondary antibodies (Jackson Immunosciences), the blots were developed using the chemiluminescence detection kit ECL-Plus (Amersham) according to the manufacturer's instructions. Quantification was performed on scanned images of blots using the Image Lab software, and the values shown on the graphs represent normalization of the protein content evaluated through lamin B1 or tubulin immunoblotting.

### Chromosomal aberrations

WRN-WT, WRN-E84A and WRN-K577M cells were treated with 100 nM CPT (Sigma-Aldrich) at 37°C for 4 h and allowed to recover for an additional 12 h. Cell cultures were incubated with colcemid (0.2 μg/ml) at 37°C for 3 h until harvesting. Cells for metaphase preparations were collected and prepared as previously reported (22). For each condition used for treatment, chromosomal aberrations were examined in Giemsa-stained metaphases under a microscope (Leica) equipped with a charge-coupled device camera (Photometrics). For each time point, at least 100 chromosomes were examined by two independent investigators and chromosomal damage scored at 100×.

### Statistical analysis

All the data are presented as the mean of at least three independent experiments. Statistical comparisons of WS or WRN-mutant cells to their relevant control were analysed by Student's *t* test or by Mann–Whitney test. P < 0.05 was considered significant. Multiple comparisons between samples were performed by the Kruskal–Wallis test. P < 0.05 was considered significant. The number of asterisks denotes the level of significance as follows: ****P < 0.0001; ***P < 0.001; **P < 0.01; *P < 0.05.

## RESULTS

### Helicase and exonuclease activities of WRN differently affect replication fork progression after CPT treatment

**Figure 1.**
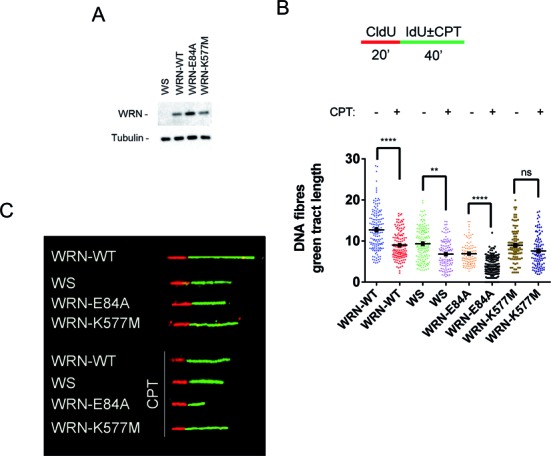
Replication fork progression on exposure to a low dose of CPT is affected by loss of WRN exonuclease activity. **(A)** WB analysis of WRN expression in WS-derived cells stably expressing the WRN wild-type (WRN-WT) and the exonuclease-dead (WRN-E84A) or helicase-dead (WRN-K577M) mutant. (**B**) Experimental scheme of dual labelling replication assay for DNA fibres. Red tract: CldU; green tract: IdU. CPT was added during the second pulse. Dot plot showing the IdU tract length (μm) of ongoing forks in single DNA fibres from WS cells, WS-derived cells stably expressing the WRN wild-type (WRN-WT), and the exonuclease-dead (WRN-E84A) or helicase-dead (WRN-K577M) mutants, in the presence (CPT) or absence (Untr) of 50 nM CPT as indicated in (A). The length of the green tracts was measured in at least 100 well isolated DNA fibres from two independent experiments. Mean values are represented as horizontal black lines ± SE (ns = not significant; *P* > 0.05; **P < 0.01; ***P < 0.001; ****P < 0.0001; Mann–Whitney test). (**C**) Images of representative DNA fibres from the different cell lines treated or not treated with CPT.

Short treatments with nanomolar concentrations of CPT determine fork reversal instead of DSBs, which are prevalent at high doses of the drug, and replication fork delay positively correlates with replication fork reversal (8). *In vitro*, both types of enzymatic activities of WRN are involved in fork reversal and restoration (19,21). To determine if both activities are equally important *in vivo*, we exposed cells expressing the wild-type form of WRN or its catalytically inactive mutants to a short treatment with a low dose of CPT and analysed replication fork progression. We utilized DNA fibre analysis to monitor replication perturbation at the single-molecule level in WRN-deficient (WS) cells and in WS-derived cells stably expressing the WRN wild-type (WRN-WT) or the mutant forms of WRN affecting either exonuclease (WRN-E84A) or helicase (WRN-K577M) activity (Figure 1A). We sequentially labelled active replication forks with CldU (red fluorescence) and IdU (green fluorescence), accompanied by treatment with 50 nM CPT during the IdU pulse (Figure 1B and Supplementary Figure S1). Progression of the ongoing forks was estimated by measuring the length of the IdU tracts (Figure 1B). In agreement with previous reports, loss of WRN affected fork progression under unperturbed conditions (26); in addition, the expression of an exonuclease- or a helicase-dead form of WRN resulted in comparably shorter IdU-labelled replication tracts (Figure 1B and C). Camptothecin treatment significantly reduced the length of the IdU-labelled nascent strand in all the cell lines, with the exception of the helicase-dead mutant (Figure 1B and C). Strikingly, loss of WRN exonuclease activity led to more pronounced reduction in the length of nascent DNA (treated/untreated ratio of the median IdU tract length = 0.69 in WRN-WT and 0.56 in WRN-E84A cells) (Figure 1B and C). The length of the nascent strand on treatment with 4 mM hydroxyurea or a high dose of CPT (5 μM) in the WRN-E84A mutant was not lesser than that in WRN wild-type cells, suggesting that the requirement for WRN exonuclease function may be prominent after treatment with low doses of CPT (Supplementary Figure S2). To test if reduced fork progression after a low dose of CPT was also observed in the absence of other enzymes involved in fork remodelling, we depleted either SMARCAL1 or RECQ1 by using RNAi in wild-type cells (Supplementary Figure S3A and D). Decrease in the levels of these enzymes involved in remodelling the stalled replication forks (9,27) reduced replication fork progression under unperturbed conditions, but did not further delay fork progression after CPT treatment (Supplementary Figure S3). In contrast, depletion of SMARCAL1 or RECQ1 led to longer replication tracts after treatment as compared to those noted for the Ctrl RNAi cells (treated/untreated ratio of the median IdU tract length = 0.62–0.67 in siCtrl, 0.89 in siSMARCAL1 cells and 0.78 in siRECQ1 cells).

These results suggest that loss of each enzymatic activity of the WRN differently affects replication fork progression after low-dose CPT treatment, and that, in the WRN mutant cells, differences in replication fork progression are unrelated to DSB accumulation.

### WRN exonuclease activity limits MRE11-dependent processing at the replication fork perturbed by CPT

**Figure 2.**
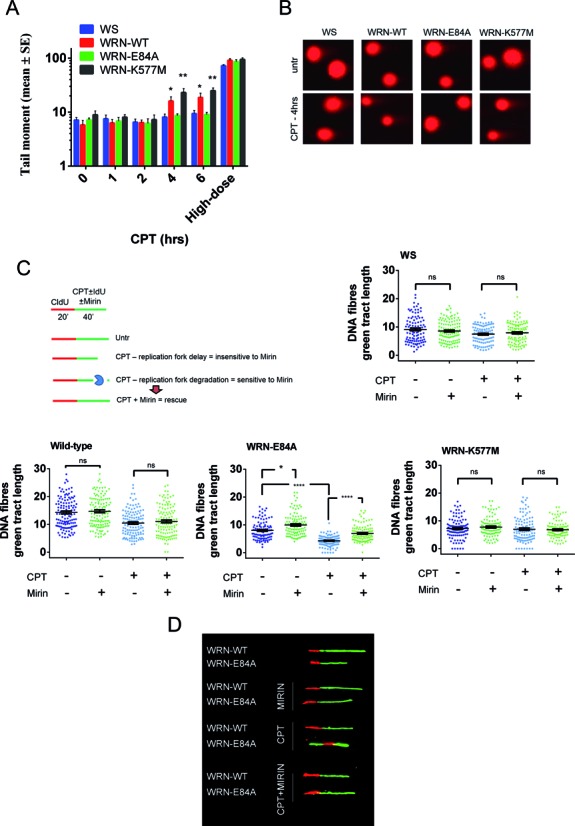
WRN exonuclease activity protects from MRE11-dependent processing of replication forks upon CPT treatment. (**A**) Analysis of DSB accumulation by the neutral comet assay. Cells were treated or not treated with 50 nM for the indicated time, or with 3 μM CPT (high-dose) for 6 h, and then subjected to the neutral comet assay. In the graph, data are presented as mean tail moment ± SE from three independent experiments (*P < 0.05; **P < 0.01 with respect to wt; Mann–Whitney test). (**B**) Representative images from the neutral comet assay are shown. (**C**) Schematic of DNA fibres experiment (left). Analysis of IdU tract length of ongoing forks (right and bottom). Dot plots show distribution of IdU tract lengths (μm) from single DNA fibres in WS cells, WS-derived cells stably expressing the WRN wild-type (WRN-WT), and the exonuclease-dead (WRN-E84A) or helicase-dead (WRN-K577M) mutants, in the presence (CPT) or absence (Untr) of 50 nM CPT. When indicated, mirin was added together with IdU, as in the experimental scheme. The length of the green tracts was measured in at least 100 well isolated DNA fibres from two independent experiments. Values are represented as means ± SE. (**D**) Images of representative DNA fibres from the WRN-WT or WRN-E84A cell lines treated or not treated with CPT and mirin (*P < 0.05; ****P < 0.0001; ns = not significant; Kruskal–Wallis test).

Our data demonstrate that loss of the WRN exonuclease greatly affected replication at a low dose of CPT. Replication fork progression after treatment could be altered by formation of DSBs or by unscheduled exonucleolytic processing of the nascent strand (13,28). To determine the mechanism underlying the enhanced reduction in the length of nascent strands observed in cells expressing an inactive WRN exonuclease, we first analysed the formation of DSBs in cells expressing the different WRN catalytic mutants. The neutral comet assay confirmed that 50 nM CPT did not induce DSBs at the time point corresponding to our replication analysis, irrespective of the status of WRN catalytic activities (Figure 2A and B). At the late time points (4 and 6 h), CPT enhanced DNA breakage only in cells expressing the wild-type or the helicase-dead form of WRN; however, the DSB level was approximately 10-fold lower than that observed at the high dose (Figure 2A and B). Since the reduction in the length of nascent strands observed in the WRN-E84A cells was unrelated with differences in DSB formation, we next verified whether shorter replication tracts correlate with unscheduled degradation of nascent strands. Indeed, perturbed replication forks can be targeted by the MRE11 exonuclease, resulting in degradation of nascent DNA and shortening of the replication tracts under a few pathological conditions (13). Interestingly, MRE11 has been recently involved in unscheduled nascent strand degradation after DSB induction at collapsed forks in the absence of WRN (29). Therefore, we analysed whether treatment with mirin, a small-molecule inhibitor of MRE11 (30), would restore the wild-type length of nascent strands on CPT treatment. Exposure to a 50 μM dose of mirin, which is sufficient to inhibit MRE11 *in vivo* (30), resulted in recovery of the length of the replication tracts to almost the wild-type level in unperturbed WRN-E84A cells (Figure 2C and D). Consistent with this, inhibition of MRE11 rescued the apparent CPT-induced replication defect in cells expressing the WRN exonuclease-dead mutant, as shown by the increased length of the IdU tracts (Figure 2C). Interestingly, mirin treatment restored the wild-type treated/untreated ratio between median IdU tract lengths (ratio = 0.69 in WRN-WT and 0.70 in WRN-E84A+mirin cells). To substantiate our results, we depleted MRE11 in WRN-WT or WRN-E84A cells by RNAi (Supplementary Figure S4A) and analysed the IdU-tract length after treatment with CPT, as described in Figure 2C. In agreement with the MRE11 inhibition by mirin, MRE11 knockdown recovered the length of the nascent strand in the WRN exonuclease mutant cells (Supplementary Figure S4B and C). Interestingly, depletion of MRE11 increased the length of the nascent strand over the wild-type level in WRN-E84A cells after CPT treatment (Supplementary Figure S4B).

These results show that reduced replication fork progression associated with loss of WRN exonuclease activity is not associated with altered formation of DSBs at perturbed forks but is the consequence of unscheduled MRE11-dependent degradation of the nascent strand. Furthermore, our findings demonstrate that MRE11 inactivation reverts the apparent slow-replicating phenotype of WRN-exonuclease defective cells under unperturbed cell growth.

### Loss of WRN exonuclease leads to increased formation of nascent ssDNA at CPT-perturbed replication forks by MRE11 and to enhanced MRE11-ssDNA association

**Figure 3.**
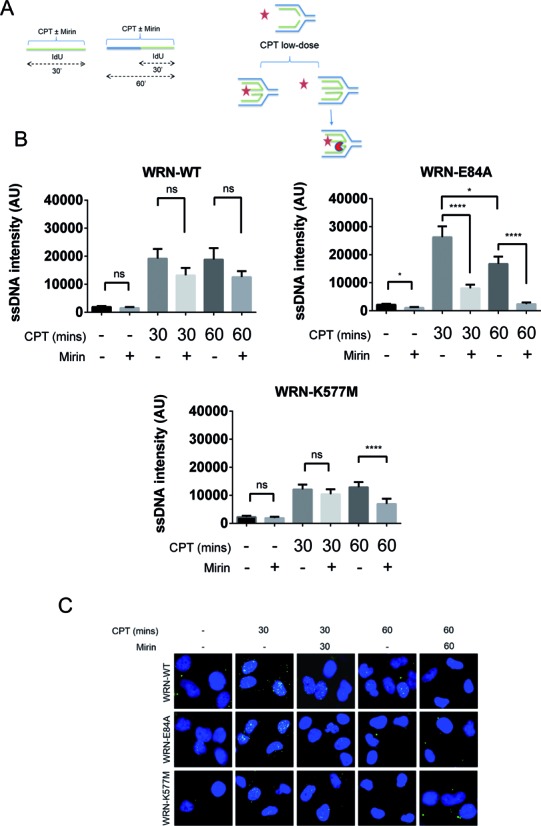

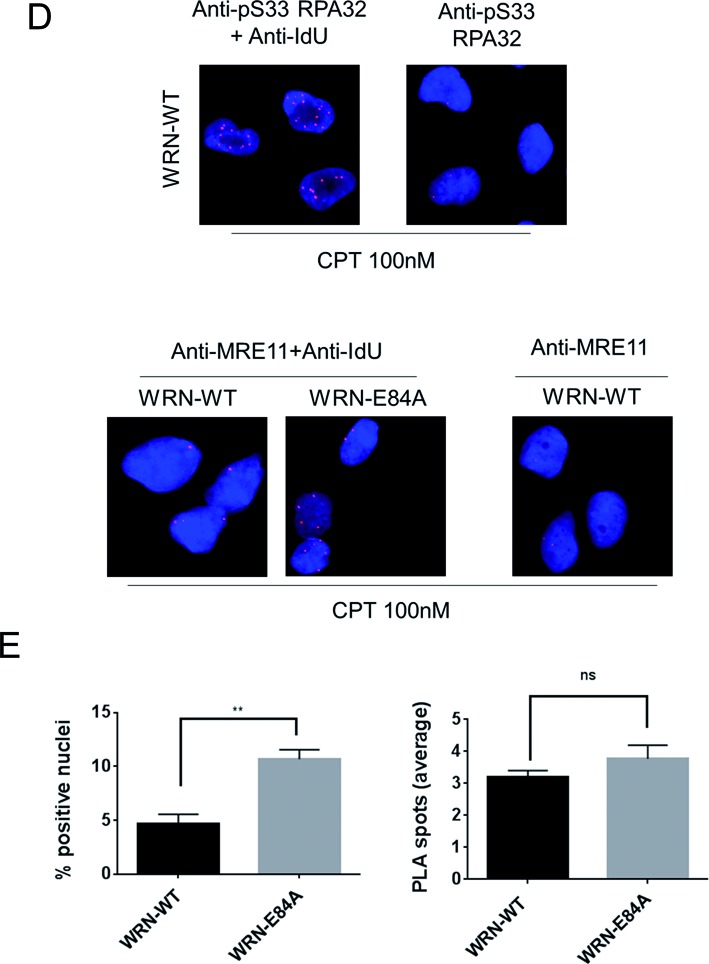
Loss of either type of catalytic activity of WRN differently affects MRE11-dependent formation of ssDNA at nascent strands after CPT treatment. (**A**) Experimental scheme and model for formation and detection of ssDNA at nascent strands by our ssDNA assay. Template DNA is depicted in blue, while nascent DNA is green. Under native conditions, anti-IdU antibodies (red star) will only label nascent DNA if it is single-stranded or degraded by exonucleases (red Pacman). (**B**) Dot plots show the mean intensity of ssDNA staining for single nuclei from cells expressing the wild-type (WRN-WT) or each catalytically dead form of WRN (WRN-E84A, WRN-K577M). Cells were either left untreated or challenged with 50 nM CPT for increasing periods, as indicated. Mirin was added together with CPT, while IdU was added in the last 30 min. The intensity of the anti-IdU immunofluorescence was measured in at least 100 nuclei from two independent experiments. Values are represented menas ± SE. (**C**) Representative images of ssDNA labelling from cells treated as those in ‘B’ (*P < 0.05; ****P < 0.0001; ns = not significant; Mann–Whitney test). (**D**) (Bottom) Representative images of PLA showing MRE11 colocalization with ssDNA in WRN-WT and WRN-E84A cells. Association of ssDNA and RPA32 phosphorylated at S33 has been used as a positive PLA control (Top). (**E**) Graphs show the percentage of positive nuclei and the number of PLA spots per cell in WRN-WT and WRN-E84A cells. Cells were treated with 100 nM CPT for 1 h. At least 300 nuclei were analysed for each experimental point and PLA spots were counted on at least 100 positive cells from two independent experiments. Values are represented as means ± SE (ns = not significant; **P < 0.01, Mann–Whitney test).

Our DNA fibre assay demonstrated that the reduced fork progression observed in the absence of the WRN exonuclease is influenced by MRE11-dependent degradation. To better understand the role of MRE11, we monitored the formation of ssDNA at nascent strands after treatment with a low dose of CPT in wild-type, WRN-E84A and WRN-K577M cells, in the presence and absence of mirin (Figure 3A). Labelling of nascent DNA with a short pulse immediately before sampling, in the absence of DSBs, allows detection of ssDNA only at paired nascent strands, and has been considered as an indirect *in vivo* assay for fork remodelling and regression (31,32). As expected, ssDNA was barely detectable under unperturbed conditions in all cell lines, irrespective of the presence of WRN or its enzymatic activities (Figure 3B and C). Replication perturbation induced by a low dose of CPT was sufficient to stimulate the formation of ssDNA at nascent strands, albeit at levels that are very low if compared to those resulting from treatment with a DSB-inducing CPT dose (Supplementary Figure S5). The amount of ssDNA induced by the low dose of CPT appeared higher in the WRN-E84A cells where it decreased with time, whereas the extent of single-stranded nascent DNA did not change with time in wild-type cells (Figure 3B and C). In contrast, the level of ssDNA was consistently lower in the WRN-helicase defective cells (Figure 3B and C).

Interestingly, mirin decreased the amount of nascent ssDNA in unperturbed WRN-E84A cells but did not affect its formation in wild-type or WRN-K577M cells (Figure 3B and C). Most importantly, inhibition of MRE11 by mirin led to a significant reduction in the amount of the nascent ssDNA detected in the WRN-exonuclease mutant cells after CPT (Figure 3B and C). In contrast, mirin failed to inhibit CPT-induced ssDNA formation in wild-type cells and WRN-K577M cells at 30 min (Figure 3B and C), suggesting that other exonucleases may be involved. Surprisingly, ssDNA formed at nascent strands in WRN-helicase deficient cells after 60 min of treatment was decreased by MRE11 inhibition. The correlation between MRE11 activity and ssDNA production in the absence of the WRN exonuclease activity was independently confirmed using immunofluorescence to detect chromatin-associated RPA32 foci after CPT treatment. In agreement with the results of the non-denaturing IdU assay, we observed that mirin did not significantly reduce RPA32 relocalization in foci in wild-type or WRN-K577M cells, while it significantly decreased their number in WRN-E84A cells (Supplementary Figure S6A and B).

Consistent with rapid activation of MRE11, at 1 h of treatment, we observed approximately 4-fold more MRE11-positive nuclei in WRN-E84A cells than in the wild-type counterpart (Supplementary Figure S6C and D). Enhanced accumulation of MRE11-positive nuclei was also observed in WRN-K577M cells, but only at 4 h of treatment (Supplementary Figure S6C and D). To functionally correlate enhanced MRE11 localization in WRN-E84A cells with ssDNA formation at the nascent strand, we used the PLA to detect association between MRE11 and ssDNA at the single-cell level. The PLA allows for *in situ* visualization of interactions between two proteins since the PLA signal is only generated if the two targets are in close proximity, that is at a distance ranging from 0 to 30 nm (33). We first validated our approach by performing PLA using an anti-pS33 RPA32 antibody and the anti-IdU antibody used for the detection of ssDNA. RPA32 phosphorylated at S33 is a marker of stalled replication forks and is a diagnostic sign of ssDNA. However, when PLA was performed with only the anti-pS33 RPA2 antibody, no signal was detectable in most nuclei, while a few shown a single PLA dot (Figure 3D). In contrast, when we performed PLA using both the anti-pS33 and the anti-IdU antibodies to detect ssDNA at nascent strands under non-denaturing conditions (Figure 3A), a strong increase in the number of PLA dots was clearly observed (mean = 15 ± 4; Figure 3D), confirming that association of a ssDNA-binding protein with IdU-labelled ssDNA can be effectively visualized by this technique. When we analysed the localization of MRE11 to nascent ssDNA after CPT treatment in WRN-WT or WRN-E84A cells, we found that the fraction of nuclei with more than two MRE11/ssDNA PLA spots was 2-fold higher in cells with mutated WRN exonuclease (Figure 3E), which is consistent with the genetic dependency of ssDNA formation showed in Figure 3B. Interestingly, the average number of MRE11/ssDNA spots did not significantly change between wild-type and WRN-E84A mutant cells (Figure 3E), probably because some of the interactions might not be detected because of steric hindrance or DNA structures.

Altogether, these findings show enhanced localization of MRE11 at ssDNA sites of nascent strands in the absence of the WRN exonuclease activity. They also demonstrate specific MRE11-dependent formation of nascent ssDNA after treatment with a low dose of CPT in cells expressing exonuclease-dead WRN but not in the wild type or in the absence of WRN-helicase activity.

### EXO1 but not DNA2 contributes to the nascent strand degradation occurring on TOP1 inhibition in the absence of WRN exonuclease activity

Our results showed that an MRE11-dependent mechanism generated a significant amount of ssDNA at nascent strands shortly after replication perturbation by a low dose of CPT, and in the absence of DSBs, when the WRN exonuclease was not functional. Therefore, we investigated whether other exonucleases, such as DNA2 and EXO1 (34), which collaborate with MRE11 during end-resection at DSBs, could be involved in the unscheduled degradation of nascent strands at CPT-perturbed replication forks.

**Figure 4.**
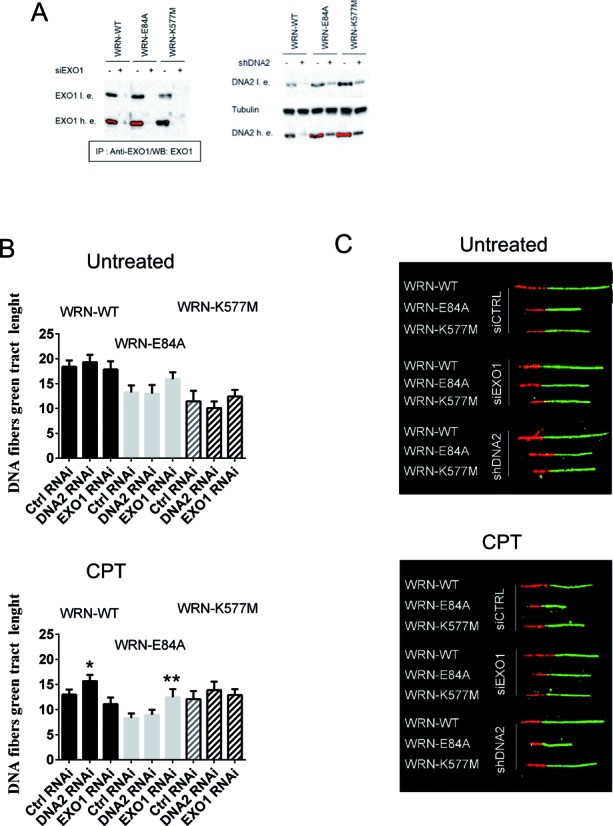
Different exonucleases affect fork elongation in CPT, depending on the WRN status. (**A**) Western immunoblotting showing depletion of DNA2 or EXO1 in cells expressing the wild-type form of WRN (WT) or its catalytically dead mutants (E84A and K577M). Whole cell extracts were prepared at 48 h after transfection or nucleofection with EXO1 siRNAs or DNA2 shRNA, respectively. The amount of EXO1 was evaluated after IP with the anti-EXO1 antibody. Lamin B1 was used as the loading control in WB with anti-DNA2 antibodies. l.e. = low exposure, h.e. = high exposure. Saturated signals are in red. (**B**) Analysis of IdU-labelled tract length of ongoing forks. The graphs show the mean value of IdU tract length (μm) from single DNA fibres in WS-derived cells stably expressing the WRN wild-type (WRN-WT) and the exonuclease-dead (WRN-E84A) or helicase-dead (WRN-K577M) mutants, in the presence (CPT) or absence (Untreated) of 50 nM CPT. Forty-eight hours before treatment, the cells were transfected with the indicated RNAi reagent. The length of the green tracks was measured in at least 100 well isolated DNA fibres from two independent experiments. Data are presented as mean ± SE (*P < 0.05; **P < 0.001 with respect to Ctrl RNAi; Kruskal–Wallis test). (**C**) Images of representative DNA fibres from the different cell lines.

We first analysed the length and integrity of the neo-synthesized DNA by using the DNA fibre assay in cells depleted of DNA2 or EXO1 by RNAi (Figure 4A). In untreated cells, downregulation of either DNA2 or EXO1 did not significantly affect the IdU tract length (Figure 4B and C). In contrast, depletion of DNA2 in wild-type cells or EXO1 in the WRN-E84A mutant significantly increased the IdU tract length after CPT, resulting in apparent rescue of the replication fork progression rate (Figure 4B and C).

**Figure 5.**
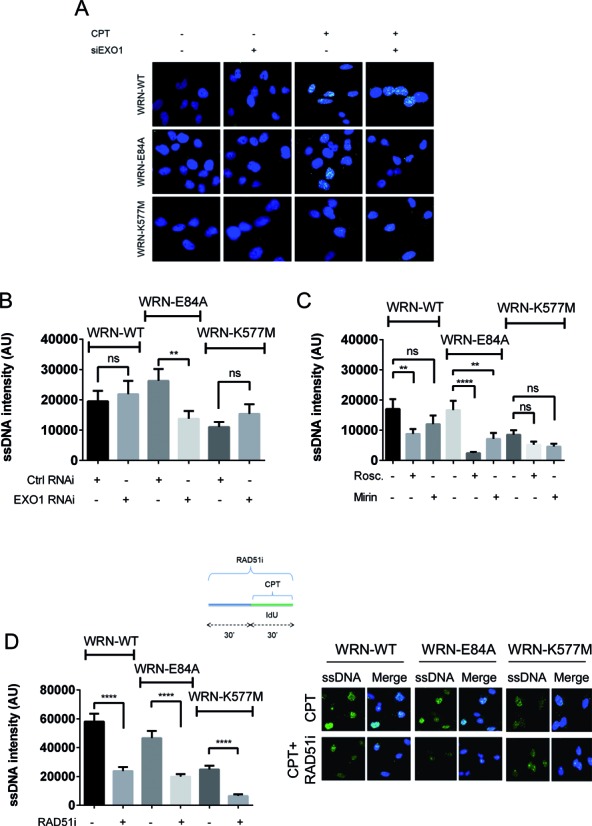
Loss of WRN exonuclease activity stimulates formation of ssDNA at nascent strands through EXO1 and CDK-dependent mechanisms, which act downstream of RAD51. Cells expressing the wild-type form of WRN (WRN-WT) or each catalytically dead form of WRN (WRN-E84A, WRN-K577M) were transfected with Ctrl siRNAs or siRNAs against EXO1 prior to exposure to 50 nM CPT for 30 min. IdU was added to cells in the last 30 min to label nascent DNA. (**A**) Representative images of ssDNA formation, as evaluated by non-denaturing anti-IdU immunostaining. (**B**) Graphs show the mean intensity of ssDNA staining for single nuclei from each cell line after treatment with 50 nM CPT for 30 min. Cells were treated with the indicated RNAi at 48 h before the ssDNA assay. The intensity of the anti-IdU immunofluorescence was measured in at least 100 nuclei from two independent experiments. Values are represented as means ± SE. (**C**) Dot plots show the mean intensity of ssDNA staining for single nuclei from each cell line after treatment with 50 nM CPT for 30 min. Roscovitine or mirin was added at 30 min before and left during CPT treatment, while IdU was added together with CPT. Intensity of the anti-IdU immunofluorescence was measured in at least 100 nuclei from two independent experiments. Values are represented as means lines ± SE. (**D**) Dot plots show the mean intensity of ssDNA staining for single nuclei from WS cells and cells expressing the wild-type (WRN-WT) or each catalytically dead form of WRN (WRN-E84A, WRN-K577M). Cells were either left untreated or challenged with 50 nM CPT for 30 min. Where indicated, cells were pretreated with the RAD51 inhibitor for 30 min before CPT. Intensity of the anti-IdU immunofluorescence was measured in at least 100 nuclei from two independent experiments. Values are represented as means ± SE. Representative images are shown (ns = not significant; **P < 0.01; ****P < 0.0001; Mann–Whitney test). siEXO1 = EXO1#1 oligo.

Since depletion of EXO1 recovered the IdU tract length in the WRN-E84A cells, we next examined the levels of ssDNA formed at nascent strands in CPT-treated wild-type, WRN-E84A and WRN-K577M cells in which EXO1 was downregulated by RNAi. Consistent with the results of the DNA fibre assay, we found that EXO1 knockdown significantly reduced accumulation of nascent ssDNA in the WRN exonuclease mutant cells, but did not affect its formation in wild-type or WRN-K577M cells (Figure 5A and B). Similar results were observed on performing the replication assay using siRNAs to deplete DNA2 and on performing the nascent ssDNA assay and transfecting cells with different RNAi oligos to knockdown EXO1 (Supplementary Figure S7).

It is known that EXO1 activity during the end-resection of DSBs is regulated by CDKs (35); therefore, we investigated whether similar regulation could take place during the nascent strand degradation occurring in WRN-E84A cells. To this end, we used roscovitine to inhibit CDKs prior to CPT treatment and analysed ssDNA formation at the nascent strand by IdU labelling. Treatment with mirin was used as a positive control. In wild-type cells, roscovitine significantly reduced the amount of ssDNA formed after treatment, while, as expected, mirin only partially reduced ssDNA formation (Figure 5C). In contrast, roscovitine completely suppressed ssDNA generated at nascent strands in WRN-E84A cells after treatment (Figure 5C). Both roscovitine and mirin did not significantly alter CPT-induced ssDNA formation in WRN-K577M cells (Figure 5C).

Our nascent ssDNA assay, in the absence of DSB formation and processing at the fork, should pinpoint degradation of paired nascent strands after fork remodelling, and probably fork regression, as suggested elsewhere (4,5,31,32). To gain mechanistic insights into the MRE11 and EXO1-dependent strand degradation occurring in the absence of WRN exonuclease activity, we investigated whether the use of an inhibitor destabilising RAD51 nucleoprotein filaments (36) could modulate ssDNA formation after CPT exposure. RAD51 has been found to be involved in protection from MRE11-dependent nascent strand degradation occurring during pathological replication, and most recently in the generation of regressed forks (13,15). We reasoned that nascent ssDNA would decrease after RAD51 inhibition if originated downstream an RAD51-dependent intermediate (37). Otherwise, RAD51 inhibition would have to lead to increase in or maintenance of the amount of nascent ssDNA, if ssDNA derived from loss of RAD51-mediated fork stabilization (12,13). As shown in Figure 5D, RAD51 inhibition led to substantial suppression of nascent strand degradation in all cell lines, irrespective of the presence of a catalytically inactive WRN protein.

Collectively, these results demonstrate that two different mechanisms target nascent strands on replication perturbation by CPT. In wild-type cells, DNA2-mediated degradation may take place and may be responsible for the apparent reduction in the replication rate. However, in cells with impaired WRN exonuclease, more robust degradation may derive from the combined CDK-regulated action of MRE11 and EXO1. Moreover, our findings suggest that MRE11 and EXO1-dependent nascent strand degradation triggered in the absence of the WRN exonuclease activity is unlikely to derive from loss of RAD51-dependent fork protection, but rather occurs downstream of RAD51-dependent fork remodelling.

### Loss of WRN catalytic activities impairs association with nascent ssDNA after TOP1 inhibition

**Figure 6.**
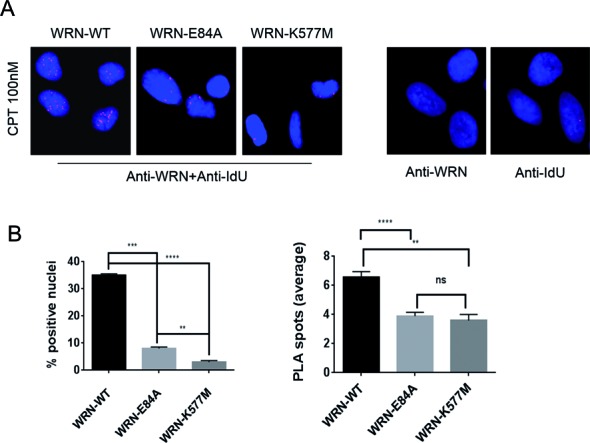
WRN is localized at nascent ssDNA in response to CPT-induced replication perturbation and this association with ssDNA is affected by mutations abrogating exonuclease or helicase activity. (**A**) Representative images of PLA showing association of WRN with nascent ssDNA (left). The right panel shows the negative controls from PLA with each single antibody. (**B**) Graphs show the percentage of PLA-positive nuclei and the number of PLA spots per cell in WRN-WT, WRN-E84A and WRN-K577M cells. Cells were treated with 100 nM CPT for 1 h. At least 300 nuclei were analysed for each experimental point and PLA spots were counted on at least 100 positive cells from two independent experiments. Values are presented as means ± SE (ns = not significant; **P < 0.01; ***P < 0.001; ****P < 0.0001; Mann–Whitney test).

We found that expression of an exonuclease-dead WRN protein determines the activation of an MRE11 and EXO1-dependent mechanism that degrades the nascent strand at perturbed forks. To further investigate the underlying mechanism, we analysed the ability of WRN and its catalytic mutants to bind nascent ssDNA *in vivo*. To this end, cells were treated with CPT for 1 h, and the WRN-ssDNA association tested with the PLA by using an anti-WRN antibody and an anti-IdU antibody under non-denaturing conditions, as shown in Figure 3. The PLA performed with single antibodies resulted in very few spots in very few cells, proving the specificity of the assay (Figure 6A, right panel). In contrast, the results of PLA performed with both the two antibodies showed that WRN was associated with nascent ssDNA in more than 30% of nuclei; the mean number of PLA spots was 6.2 (Figure 6A and B). Interestingly, both the WRN catalytic mutants showed significant reduction in the number of PLA-positive nuclei, as well as in the number of PLA spots per nucleus (Figure 6A and B).

These results indicate that WRN is recruited at nascent ssDNA in response to CPT-induced replication perturbation and that each type of catalytic activity is important for maintaining protein binding at the ssDNA region of nascent strands.

### MRE11-dependent processing of nascent strands is responsible for poor fork recovery in the absence of WRN exonuclease activity

**Figure 7.**
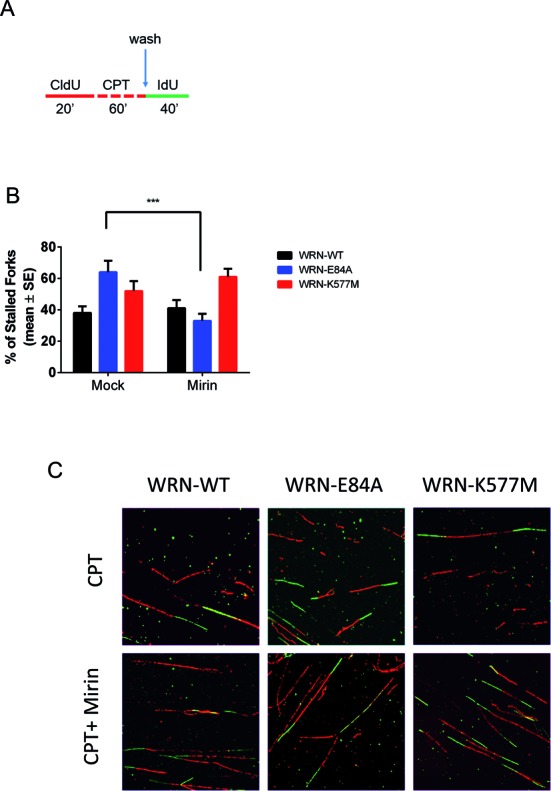
MRE11-dependent degradation of the nascent strand impairs replication fork recovery in cells expressing the exonuclease-dead form of WRN. (**A**) Experimental scheme of dual-labelling replication recovery assay for DNA fibres. Red tract: CldU; green tract: IdU. (**B**) The graph shows the average number of stalled forks after recovery from treatment with a low dose of CPT, as described in (A). Where indicated, mirin was added to cultures together with CPT and during the IdU pulse. Data are presented as mean ± SE from three independent experiments. A minimum of 50 CldU-positive tracks were analysed in each experimental point (***P < 0.001 with respect to mock inhibition; Student's *t*-test). (**C**) Images of representative DNA fibres from the different cell lines.

In response to replication perturbation induced by a low dose of CPT, cells expressing an inactive WRN exonuclease engaged MRE11 and EXO1 to degrade the nascent strand, probably during RAD51-dependent fork remodelling. To test whether this mechanism contributes to fork restart in the absence of a functional WRN exonuclease, we analysed replication fork recovery after CPT treatment using DNA fibres. Experiments were performed in wild-type, WRN-E84A and WRN-K577M cells using a modified double-labelling protocol to evaluate fork recovery after removal of CPT (Figure 7A). Under such conditions, approximately 40% of forks were stalled in wild-type cells, while their proportion ranged between 60% and 50% in WRN-E84A and WRN-K577M cells, respectively (Figure 7B). Concomitant treatment with CPT and mirin did not affect the fraction of stalled forks in wild-type cells as well as in WRN-K577M cells, but significantly reduced the number of inactive forks in WRN-E84A cells (Figure 7C).

These findings suggest that the MRE11 and EXO1-dependent degradation of the nascent strand is a pathological event that is restrained by the WRN exonuclease activity and is mechanistically linked to poor replication fork recovery from CPT-induced fork arrest in WRN-E84A cells.

### Loss of WRN exonuclease or helicase activity bypasses PARP-mediated RECQ1 inhibition after CPT treatment

**Figure 8.**
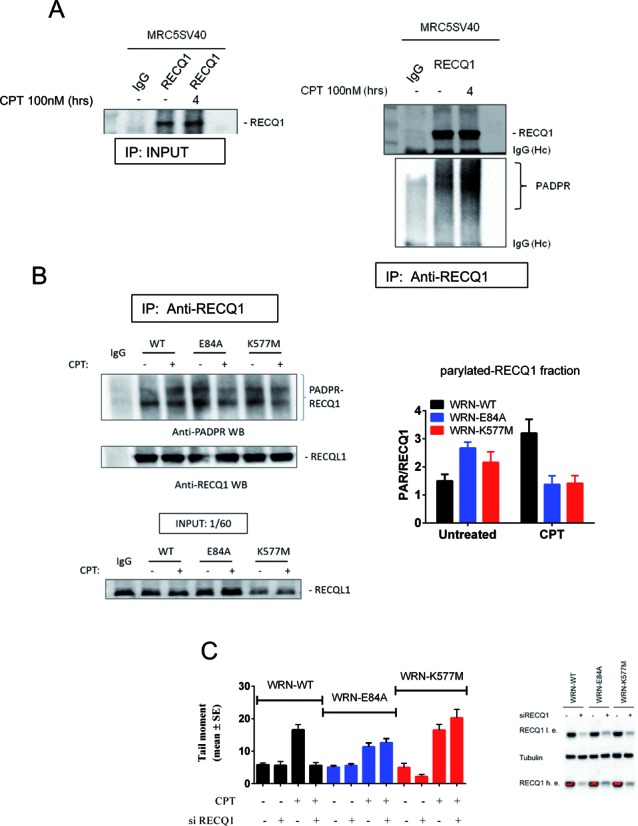
Loss of the WRN exonuclease or helicase reduces the requirement for PARP-mediated RECQ1 inhibition after CPT treatment. (**A**) CPT-induced PARylation of RECQ1 was analysed by IP/WB in MRC5SV40 cells treated with 100 nM CPT as indicated. RECQ1 or control (IgG) immunoprecipitates were separated by SDS-PAGE and sequentially analysed by western immunoblotting using anti-PAR (PADPR) and anti-RECQ1 antibodies. One-sixtieth of the total lysates was used as input. IgG (Hc): IgG heavy chains. (**B**) CPT-induced PARylation of RECQ1 was analysed by anti-RECQ1 IP/anti-PAR WB in cells expressing the wild-type (WT) or the exonuclease-dead (E84A) or helicase-dead (K577M) form of WRN, which were treated (+) or not treated (−) with 100 nM CPT for 4 h. IgG: control immunoprecipitation using WRN wild-type lysates. The graph shows quantification of RECQ1 PARylation levels from three independent western blots. IgG (Hc): IgG heavy chains. (**C**) Analysis of DSB accumulation by the neutral comet assay. Cells were either transfected with Ctrl (−) or with RECQ1 siRNAs (+); 48 h later, they were treated (+) or not with 100 nM for 6 h prior to the neutral comet assay. The graph shows data presented as mean tail moment ± SE from three independent experiments. The right panel show the knockdown efficiency, as analysed by WB. Cells expressing the wild-type (WRN-WT) or the two catalytic mutants of WRN were either transfected with Ctrl (−) or with RECQ1 siRNAs (+); 48 h later, they were analysed by WB using the indicated antibodies. l.e. = low exposure, h.e. = high exposure. Saturated signals are in red.

After treatment with a low dose of CPT, regressed forks are targeted by RECQ1, which undergoes inhibitory PARylation to avoid unscheduled fork restoration and recovery (9). To test whether the defective replication recovery observed in the WRN exonuclease mutant correlated with RECQ1 inhibition, we treated wild-type, WRN-E84A and WRN-K577M cells with a low dose of CPT and analysed PARylation of RECQ1 by immunoprecipitation and Western blotting with an anti-PAR antibody. Our analysis revealed robust accumulation of PARylated RECQ1 in wild-type cells treated with CPT (Figure 8A and B). Elevated PARylation of RECQ1 was observed in unperturbed WRN mutant cells but PARylation was substantially reduced after CPT treatment (Figure 8B).

RECQ1 PARylation prevents unscheduled fork restoration and DSB formation (9). To determine if the few DSBs detected in our WRN catalytic mutants after treatment with a low dose of CPT (Figure 2A) would depend upon reduced RECQ1 PARylation and premature activation, cells were transfected with siRNAs directed against GFP (Ctrl) or RECQ1 (siRECQ1), exposed to a low dose of CPT, and subjected to the neutral comet assay. As shown in Figure 8C, RECQ1 depletion did not affect DSB levels in untreated wild-type or WRN-E84A cells, but reduced their formation in the WRN-K577M cells. As expected, formation of CPT-induced DSBs was suppressed by RECQ1 depletion in wild-type cells (Figure 8C). Surprisingly, however, DSBs were not reduced by RECQ1 knockdown in the WRN mutant cells (Figure 8C).

Interestingly, the few DSBs detected after treatment with a low dose of CPT were reduced by the inhibition of RAD51, irrespective of the presence of the WRN helicase or exonuclease activity (Supplementary Figure S8).

These results indicate that loss of the WRN exonuclease or helicase activity makes the inhibition of RECQ1 on CPT exposure dispensable. They also show that DSBs arising after treatment with a low dose of CPT are formed through an RAD51-dependent mechanism, irrespective of the presence of an active WRN protein.

### WRN exonuclease and helicase activities at CPT-perturbed replication forks are essential to limit genome instability upon replication perturbation by low doses of CPT

**Figure 9.**
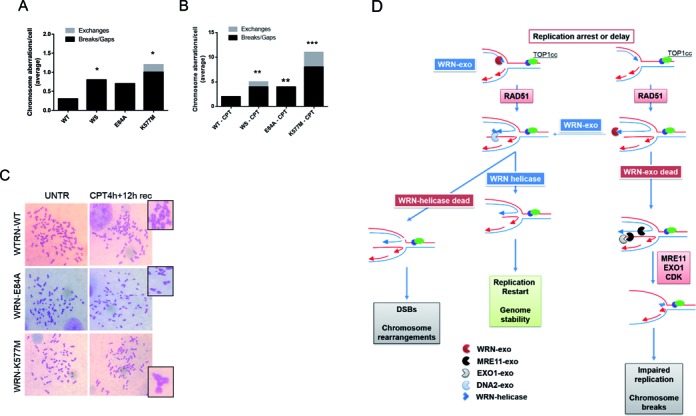
Loss of the WRN helicase but not of the WRN exonuclease greatly increased chromosome instability after treatment with low doses of CPT. (**A**,**B**) Graphs show the average number of the indicated chromosomal aberrations per cell in wild-type (WRN-WT), WS, WRN-E84A and WRN-K577M cells, under untreated conditions (A) or after 4 h of exposure to 50 nM CPT followed by 12 h of recovery in drug-free medium before harvesting (B). Data are presented as the mean of three independent experiments. Error bars are not included but SE were <15% of the mean (*P > 0.05; **P < 0.01; ***P < 0.001; Student's *t*-test). (**C**) Representative Giemsa-stained metaphases from WRN-WT, WRN-E84A and WRN-K577M cells are shown. The enlarged insets indicate characteristic chromosomal aberrations. (**D**) Proposed model of replication fork processing by WRN exonuclease or helicase activity. Replication perturbation by low doses of CPT leads to RAD51-mediated fork regression. The WRN exonuclease might trim the nascent leading strand in the 3′-5′ direction, enlarging the gap at the fork and facilitating the formation of a 5′-overhang at the extruded nascent strand. Alternatively, WRN-exo activity might trim the nascent strands once after fork regression. The action of WRN-exo would make the extruded strands ‘resistant’ to MRE11-EXO1, facilitating further processing by the WRN-DNA2 complex. In the presence of an exonuclease-dead WRN, MRE11 and EXO1 are involved in resetting of the extruded strands and preventing fork restart. Undereplicated regions could contribute to chromosome breaks, as seen in cells with defective WRN-exo activity. In cells expressing the helicase-dead WRN, DNA2 cannot process the regressed forks that are cleaved, generating DSBs and rearrangements (radial chromosomes).

Our experiments showed that loss of the WRN exonuclease or helicase activity led to significant changes in how perturbed replication forks were handled and probably caused a pathway switch in the processing of regressed forks. WRN-deficient cells are hypersensitive to CPT-induced chromosomal damage (38), and we wanted to analyse whether activation of alternative mechanisms acting at perturbed forks resulted in genome instability. Therefore, we pulse-treated wild-type, WS and WRN-mutant cells with a low dose of CPT, and chromosomal damage was analysed on metaphase spreads. Enhanced formation of chromosomal aberrations was observed in untreated WRN-E84A and WRN-K577M cells, similar to that previously seen in WS cells under unperturbed cell growth (Figure 9A). Short treatment with a low dose of CPT increased the number of chromosome gaps and breaks in wild-type cells; this number became 2-fold greater in WS or WRN-E84A cells and 4-fold greater in WRN-K577M cells (Figure 9B and C). Interestingly, while loss of WRN exonuclease increased chromosome gaps and breaks, loss of WRN, and more strikingly of WRN helicase, also led to accumulation of chromatid exchanges (Figure 9B and C).

Together, these findings demonstrated that, although WRN deficiency is correlated with chromosome instability, loss of the WRN exonuclease or helicase function leads to unscheduled activation of distinct mechanisms for processing perturbed forks, which differently affect genome instability.

## DISCUSSION

Here, we report that loss of WRN exonuclease or helicase activity activates different and alternative mechanisms of fork processing upon replication on exposure to low doses of CPT. In the absence of WRN exonuclease activity, the nascent strands undergo extensive degradation, as evidenced by shortening of replication tracts and accumulation of ssDNA at newly replicated DNA. Such degradation occurs through the combined action of MRE11 and EXO1 and is sensitive to CDK inhibition, reminiscent of end-resection at DSBs (34,39,40).

In mammalian cells, MRE11-dependent degradation of nascent strands is associated with loss of the BRCA2-FA pathway (12,13), suggesting that multiple factors concur to prevent activation of pathological processing at perturbed forks. In yeast, EXO1-dependent degradation at stalled forks prevents accumulation of reversed forks in the absence of a checkpoint response (10). Our results are consistent with a similar function of the MRE11/EXO1 exonucleases in human cells. Indeed, formation of nascent ssDNA, in the absence of replication fork breakage and resection of template DNA at the break sides, has been correlated with remodelling of the fork and likely with fork reversal (31,32). These reports, together with the observation that low doses of CPT (up to 100 nM) led to fork arrest and reversal, strongly support the possibility that processing of regressed forks is the basis of the observed nascent-strand degradation by MRE11.

In BRCA2-defective cells, MRE11-dependent degradation is irrelevant for replication fork recovery (13); however, MRE11 inhibition and depletion improve replication fork recovery in cells expressing the exonuclease-dead form of WRN, suggesting that MRE11/EXO1-dependent nascent strand degradation is a genuine pathological pathway. Notably, MRE11 inhibition in WRN exonuclease-dead mutant cells rescued the length of replication tracts also under unperturbed replication, indicating that the phenotype of apparently delayed replication associated with WRN exonuclease deficiency actually originates from unscheduled nascent strand degradation. MRE11/EXO1-dependent degradation of nascent strands in WRN exonuclease-dead cells does not overtly increase chromosome rearrangements after CPT treatment, while degradation of the stalled fork by MRE11 is responsible for the gross chromosomal damage associated with BRCA2 deficiency (13). Thus, it is likely that MRE11 can define two distinct pathological mechanisms of nascent strand processing, depending on the intermediate formed at blocked replication forks. In addition to the pathological role of MRE11 at distressed forks, it has also been found to be involved in their recovery (25,41,42), suggesting that limited MRE11-dependent degradation could be beneficial for subsequent processing at regressed forks, for instance by SMARCAL1 (27). WRN and SMARCAL1 could cooperate in fork remodelling, as proposed in an earlier study (43). We did not observe nascent strand degradation after CPT treatment in cells depleted of SMARCAL1; however, SMARCAL1-depleted and WRN-helicase mutant cells showed a similar decrease in fork delay, thereby suggesting cooperation between the helicase activity of WRN and SMARCAL1.

Interestingly, a very recent report showed that WRN, but not its catalytic activities, is required to prevent MRE11 from degrading nascent strands at replication-dependent DSBs formed after treatment with a high dose of CPT (29). WRN would counteract MRE11-dependent degradation at collapsed replication forks through the association with NBS1 and by promoting RAD51 binding to forks, a function that is also regulated by ATM-dependent phosphorylation of WRN (18). Thus, WRN could limit excessive nascent strand degradation by at least two distinct mechanisms, depending on whether DSBs are formed at perturbed forks. In their report, Su *et al*. (29) failed to detect nascent strand degradation in response to a low dose of CPT; however, our results are not necessarily contradictory. Indeed, Su *et al*. analysed nascent strand degradation after 5 h of treatment, while we tested for degradation very early (<2 h) when DSBs are absent. Moreover, we observed that ssDNA formation decreased over time in the WRN exonuclease mutant. Consistently, it is possible that early and late responses to a low dose of CPT are mechanistically distinct.

How could the WRN exonuclease protect nascent strands from MRE11-dependent degradation? One possible scenario is that WRN exonuclease activity contributes to degradation of the leading nascent strand, thus enlarging the gap for subsequent fork regression, as observed *in vitro* (19,21). The regressed fork produced after trimming by WRN exonuclease activity could have a nascent strand conformation resistant to MRE11-dependent degradation (Figure 9D). Alternatively, the enlarged gap would support RAD51 loading and fork protection, thereby counteracting fork regression (5,13). WRN and RAD51 have been found to be involved in a common pathway of replication fork recovery (44), and RAD51 filament destabilization has been shown to underlie nascent strand degradation in BRCA2-defective cells (13). However, surprisingly, we observed that RAD51 inhibition prevented ssDNA formation, irrespective of the WRN status, suggesting that, at least upon treatment with low doses of CPT, RAD51 may contribute to formation of the substrate for DNA2, MRE11 and EXO1 exonucleases, most likely the regressed fork (Figure 9D), as recently reported (15). Another attractive hypothesis is that WRN exonuclease activity contributes to the initial end-processing of regressed forks, perhaps through its interaction with MRE11 (25). WRN is a substrate of the replication checkpoint kinase ATR (18), and it is tempting to speculate that checkpoint-mediated WRN phosphorylation may be involved in preventing unscheduled nascent strand degradation, which would help explain the replicative phenotype of cells expressing the unphosphorylable WRN mutants (18,22).

If, on one hand, the WRN exonuclease is essential for preventing unscheduled MRE11 and EXO1-dependent degradation, probably because of the extruded nascent strand of a regressed fork, then, on the other hand, more limited degradation at nascent strands also takes place in wild-type cells, but with a completely different genetic dependency. Indeed, the shorter replication tracts observed after treatment in wild-type cells were not rescued after MRE11 or EXO1 inactivation, but by DNA2 depletion. In yeast and in higher eukaryotes, DNA2 and RecQ helicases, such as BLM or WRN, have been found to cooperate during end-resection (39,45,46). Interestingly, a recent report from Vindigni's group demonstrates that, upon HU treatment, the WRN helicase assists DNA2 during end-processing at paired nascent strands of regressed forks, through unwinding of the substrate (14). Our data showed that loss of the WRN helicase activity resulted in DSBs and extensive genome instability upon treatment with low doses of CPT. Although we did not observe increased formation of DSBs at early time points after treatment with a nanomolar concentration of CPT, consistent with previous reports (8,9), some DNA breakage was observed at 4 and 6 h, and was more apparent in the helicase-dead WRN mutant. Our data suggest that the loss of WRN-DNA2 axis leads to fork breakage and that the subsequent error-prone DSB repair might underlie the formation of radial chromosomes (Figure 9D), as reported elsewhere (47). DSBs formed in the absence of the WRN helicase activity are not RECQ1-dependent, and RECQ1 is not PARylated. RECQ1 PARylation, which is correlated with inhibition at regressed forks on CPT (9), is required to prevent DSBs. Thus, the absence of RECQ1 PARylation and concomitant formation of DSBs after CPT are apparently at odds. A likely explanation is that increase in PARylated RECQ1 was not seen in the WRN helicase mutant, and in the exonuclease mutant as well, because the RECQ1 substrate was ‘reset’ through alternative mechanisms after CPT treatment (Figure 9D).

In conclusion, our findings contribute to unmasking a previously unappreciated function of the WRN exonuclease in preventing pathological nascent strand degradation at regressed forks in the absence of significant amount of DSBs. Moreover, we identified a role for MRE11 and EXO1 in mediating the exonucleolytic processing of paired nascent strands under pathological replication, which takes place after treatment with low doses of CPT. Finally, we also identified a role of the WRN helicase in counteracting genome instability under conditions of fork regression, thus showing that both the enzymatic activities of WRN can play distinct roles in replication fork recovery.

More generally, this study contributes to our knowledge of normal and pathological mechanisms activated at perturbed replication forks, which are highly relevant for understanding how genomic instability can arise in genetic diseases and cancer.

## Supplementary Material

SUPPLEMENTARY DATA
