# Supplementary material for: The WRN exonuclease domain protects nascent strands from pathological MRE11/EXO1-dependent degradation
Source: Nucleic Acids Res. 2015 Aug 14;43(20):9788–803. doi: 10.1093/nar/gkv836 (PMC4787784; doi:10.1093/nar/gkv836)
Supplement: SUPPLEMENTARY DATA [file supp_43_20_9788__index.html]

The WRN exonuclease domain protects nascent strands from pathological MRE11/EXO1-dependent degradation — The WRN exonuclease domain protects nascent strands from pathological MRE11/EXO1-dependent degradation — SUPPLEMENTARY DATA 

# The WRN exonuclease domain protects nascent strands from pathological MRE11/EXO1-dependent degradation

## SUPPLEMENTARY DATA

- SUPPLEMENTARY DATA
- SUPPLEMENTARY DATA
