# Supplementary material for: The WRN exonuclease domain protects nascent strands from pathological MRE11/EXO1-dependent degradation
Source: Nucleic Acids Res. 2015 Aug 14;43(20):9788–803. doi: 10.1093/nar/gkv836 (PMC4787784; doi:10.1093/nar/gkv836)
Supplement: SUPPLEMENTARY DATA [file supp_gkv836_nar-00551-v-2015-File013.docx]

SUPPLEMENTARY FIGURE LEGENDS

Figure S1. Images of DNA fibres visualised by immunofluorescence. Pictures in the panel are from representative fields acquired with a 60x objective.

Figure S2. WRN exonuclease function may be prominent after treatment with low doses of CPT. (**A**) Schematic of DNA fibres experiment. (**B**) Images of representative DNA fibres from the different cell lines treated or not as indicated. (**C**) Analysis of IdU tract length of ongoing forks. The graphs show the mean value of IdU tract lengths (µm) from single DNA fibres in WS, WS-derived cells stably expressing the wild-type WRN (WRN-WT), its exonuclease-dead (WRN-E84A) or helicase-dead (WRN-K577M) mutants, in the presence or absence of replication-perturbing treatments as indicated. Data are presented as mean±SE. (ns = not significant p > 0.05; ** = p < 0.01, **** = p < 0.0001, Mann-Whitney test).

Figure S3. Depletion of either SMARCAL1 or RECQ1 did not reduce the length of the IdU-labelled nascent strand over that observed in the Ctrl RNAi cells. (**A**) Western immunoblotting showing depletion of SMARCAL1 in cells expressing the wild-type form of WRN (WRN-WT). Whole cell extracts were prepared at 48h after transfection with SMARCAL1 siRNA. Tubulin was used as loading control. (**B**) Analysis of IdU tract length of ongoing forks. The graph show the mean value of IdU tract lengths (µm) from single DNA fibres in WS-derived cells stably expressing the WRN wild-type (WRN-WT), treated or not with CPT, as indicated. Forty-eight hours before treatment cells were transfected with the indicated RNAi reagent. The length of the green tracks was measured in at least 100 well-isolated DNA fibres from two independent experiments. Data are presented as mean±SE. (**C**) Images of representative DNA fibres from the WRN-WT transfected or not with the indicated RNAi reagents and treated with CPT. (**D**) Western immunoblotting showing depletion of RECQ1 in cells expressing the wild-type form of WRN (WT). Whole cell extracts were prepared at 48h after transfection with RECQ1 siRNA. Tubulin was used as loading control. (**E**) Dot plots show distribution of IdU tract lengths (µm) from single DNA fibres WS-derived cells stably expressing the WRN wild-type (WRN-WT), treated or not with CPT, as indicated. The length of the green tracks was measured in at least 100 well-isolated DNA fibres from two independent experiments. Data are presented as mean±SE. (**F**) Images of representative DNA fibres from the WRN-WT cell lines treated as indicated. (*** = p < 0.001, **** = p < 0.0001, Mann-Whitney test. Where otherwise not indicated differences are not significant).

Figure S4. MRE11 knock-down recovers the length of the nascent strand in WRN exonuclease mutant cells. (**A**) Western immunoblotting showing depletion of MRE11 in WS-derived cells stably expressing the WRN wild-type (WRN-WT) or its exonuclease-dead (WRN-E84A) mutant. Whole cell extracts were prepared at 48h after transfection with MRE11 siRNA. Tubulin was used as loading control. (**B**) Analysis of IdU tract length of ongoing forks. The graph show the mean value of IdU tract lengths (µm) from single DNA fibres in WS-derived cells stably expressing the WRN wild-type (WRN-WT) or its exonuclease-dead (WRN-E84A) mutant treated or not with 50 nM CPT for 1h. Cells were transfected with the indicated RNAi reagent 48h before treatment. The length of the green tracks was measured in at least 100 well-isolated DNA fibres from two independent experiments. Data are presented as mean±SE. (**C**) Images of representative DNA fibres from the WRN-WT and its exonuclease-dead (WRN-E84A) mutant cell lines treated with CPT and silenced or not for MRE11. (ns = not significant p > 0.05; **** = p < 0.0001, Mann-Whitney test).

Figure S5. Analysis of ssDNA formation at nascent strand after treatment with low and high dose of CPT. (**A**) Experimental scheme for nascent strand ssDNA assay. (**B**) Representative images of ssDNA labelling from cells treated or not with CPT as indicated. (**C**) The graph shows the mean intensity of ssDNA staining for single nuclei from cells expressing the wild-type (WRN-WT) or each catalytically-dead form of WRN (WRN-E84A, WRN-K577M). Cells were either left untreated or challenged with CPT as indicated. IdU was added 15 minutes before with CPT addition. The intensity of the anti-IdU immunofluorescence was measured in at least 50 nuclei from two independent experiments. Mean values are represented as horizontal black lines±SE. (ns = not significant p > 0.05; *** = p < 0.001, Mann-Whitney test).

Figure S6. Analysis of RPA and MRE11 foci in WRN catalytic mutants after CPT treatment. (**A**) WS cells expressing the wild-type or each catalytically-dead WRN protein were either left untreated or challenged with 50 nM CPT for 2 h prior to anti-RPA32 immunostaining; when indicated mirin was added together with CPT to inhibit MRE11. The graph shows the number of RPA32-positive (RPA +) nuclei for each condition. Data are presented as mean ± SE from three independent experiments. (**B**) Representative images of WRN-E84A cells treated with CPT alone or in combination with mirin and immunostained with an anti-RPA32 antibody. (**C**) Cells were treated with 50 nM CPT for the indicated time prior to immunofluorescence analysis using anti-MRE11 antibody. The graph shows the number of MRE11-positive (MRE11 +) nuclei for each cell lines expressed as fold-increase over the mean untreated value. Data are presented as the mean of two independent experiments. Error bars are not shown but are < 15% of the mean. (**D**) Representative images of cells immunostained with an anti-MRE11 antibody. (* = p < 0.05, ** = p < 0.01, Student’s t-test).

Figure S7. DNA2 knock-down recovered the length of the nascent strand in the WRN-WT cells while EXO1 knock-down reduced accumulation of nascent ssDNA in the WRN exonuclease mutant cells. (**A**) Western immunoblotting showing depletion of DNA2 in WS-derived cells stably expressing the WRN wild-type (WRN-WT) or its exonuclease-dead (WRN-E84A) mutant. Whole cell extracts were prepared at 48h after transfection with DNA2 siRNA. Tubulin was used as loading control. (**B**) Analysis of IdU tract length of ongoing forks. The graph show the mean value of IdU tract lengths (µm) from single DNA fibres in WS-derived cells stably expressing the WRN wild-type (WRN-WT) or its exonuclease-dead (WRN-E84A) mutant in the presence (CPT) or absence (Untr) of 50 nM CPT. The length of the green tracks was measured in at least 100 well-isolated DNA fibres from two independent experiments. Data are presented as mean±SE. (**C**) Images of representative DNA fibres from the WRN-WT and its exonuclease-dead (WRN-E84A) mutant cell lines, transfected or not with the siDNA2 oligo and treated or not with CPT. (**D**) The graph shows the mean intensity of ssDNA staining for single nuclei from cells expressing the wild-type (WRN-WT) or its exonuclease-dead (WRN-E84A) mutant, transfected or not with the indicate siRNAs. Cells were either left untreated or challenged with CPT 100nM for 30min, as indicated. Mirin and IdU were added together with CPT, The intensity of the anti-IdU immunofluorescence was measured in at least 100 nuclei from two independent experiments. Mean values are represented as horizontal black lines±SE. (ns = not significant p > 0.05; * = p < 0.05 **** = p < 0.0001, Mann-Whitney test). EXO1 siRNA = EXO1#2.

Figure S8. Loss of the WRN helicase activity determines formation of DSBs that are RAD51-dependent after CPT. Cells were exposed to the RAD51 inhibitor (RAD51i) 30 min before being challenged with CPT as indicated. Dot plots show tail moment values from at least 50 comets and present the mean tail moment ± SE. The experiment is representative from three independent biological replicates.
