# Supplementary figures and images for: The WRN exonuclease domain protects nascent strands from pathological MRE11/EXO1-dependent degradation
Source: Nucleic Acids Res. 2015 Aug 14;43(20):9788–803. doi: 10.1093/nar/gkv836 (PMC4787784; doi:10.1093/nar/gkv836)

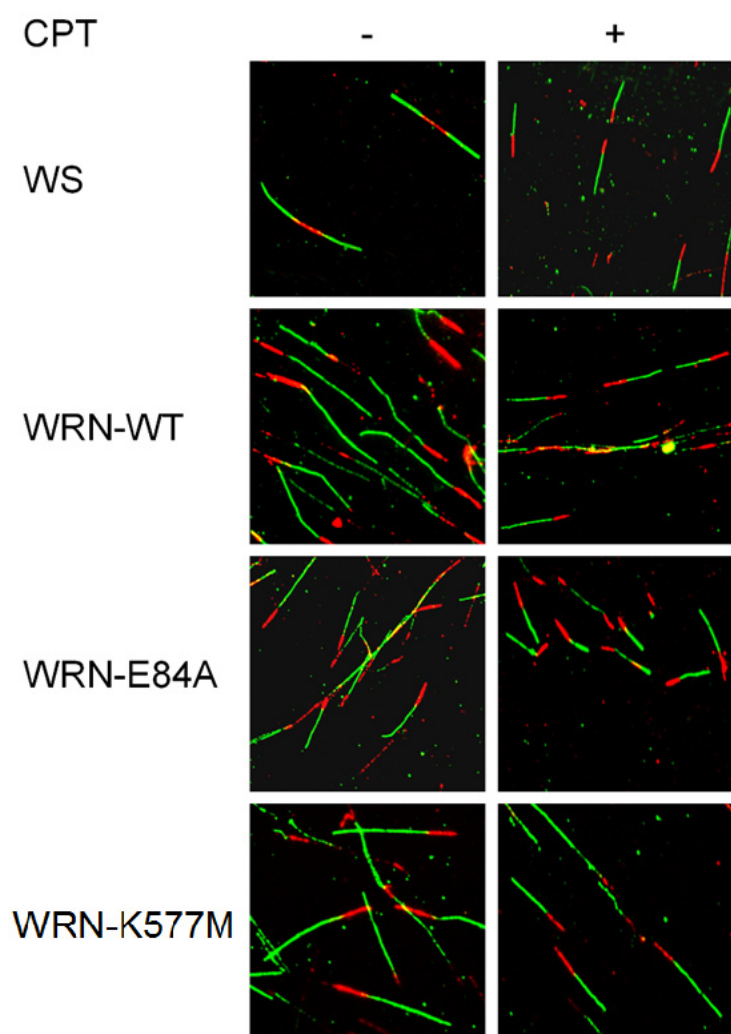

Figure S1

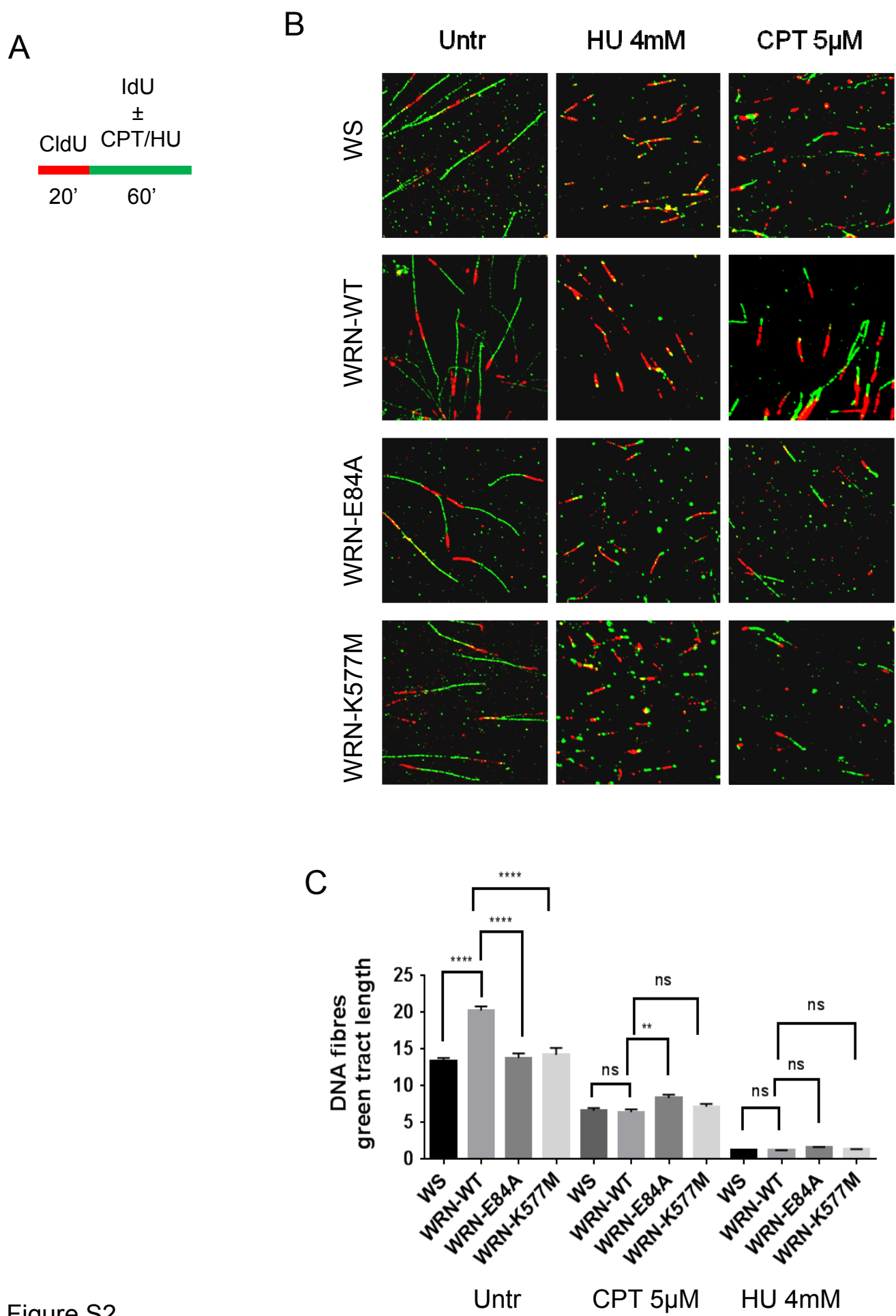

Figure S2

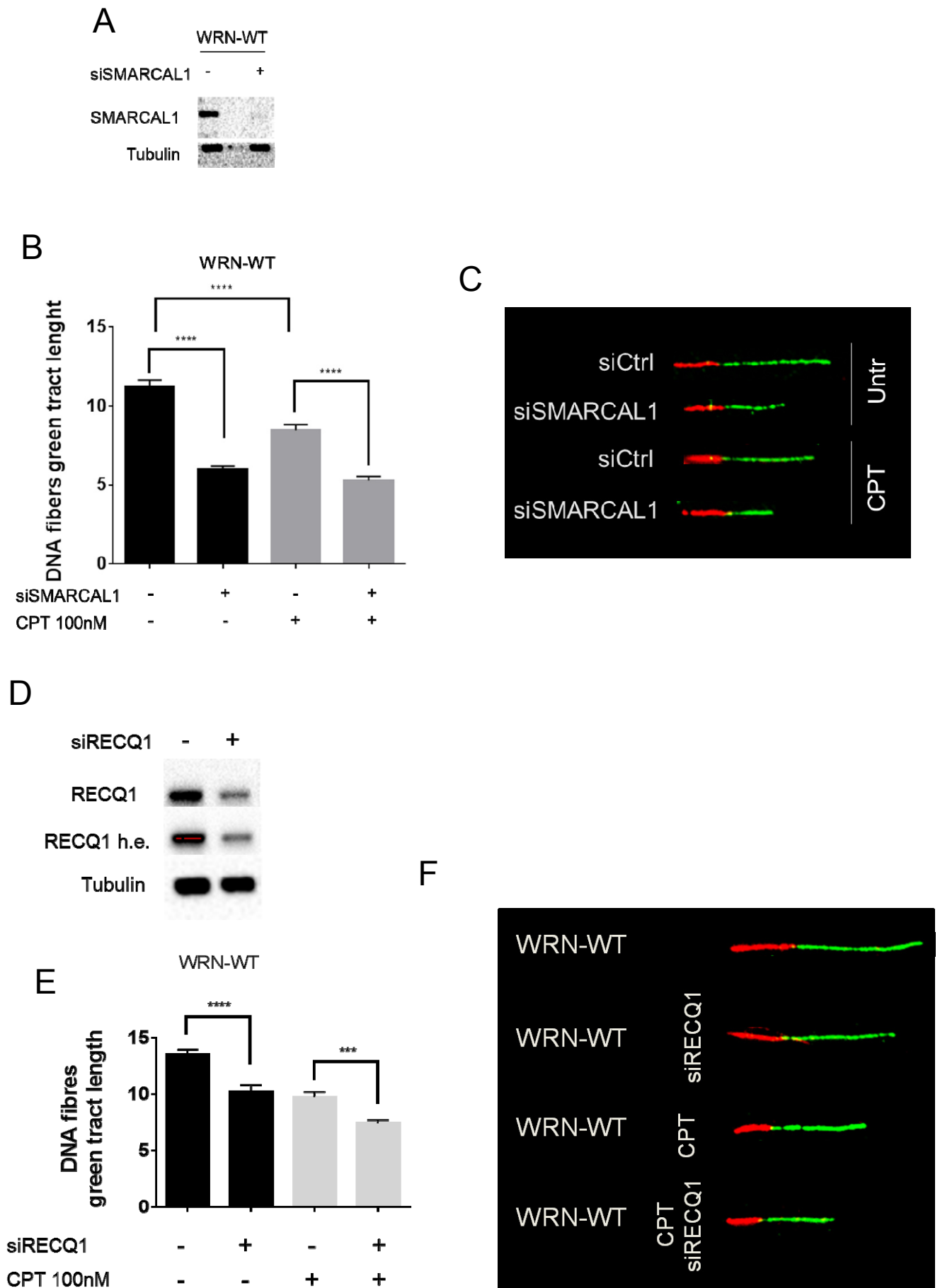

Figure S3

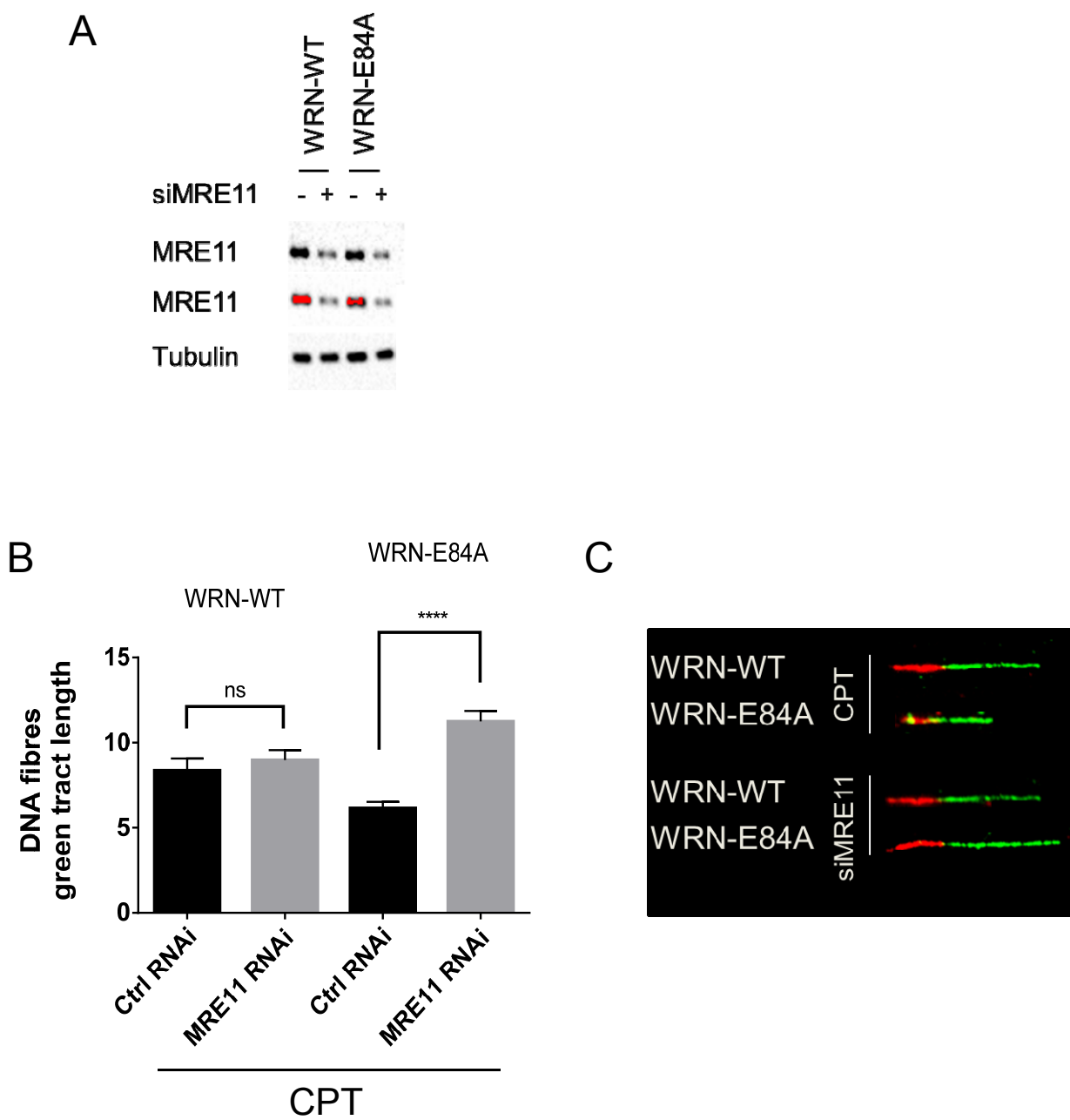

Figure S4

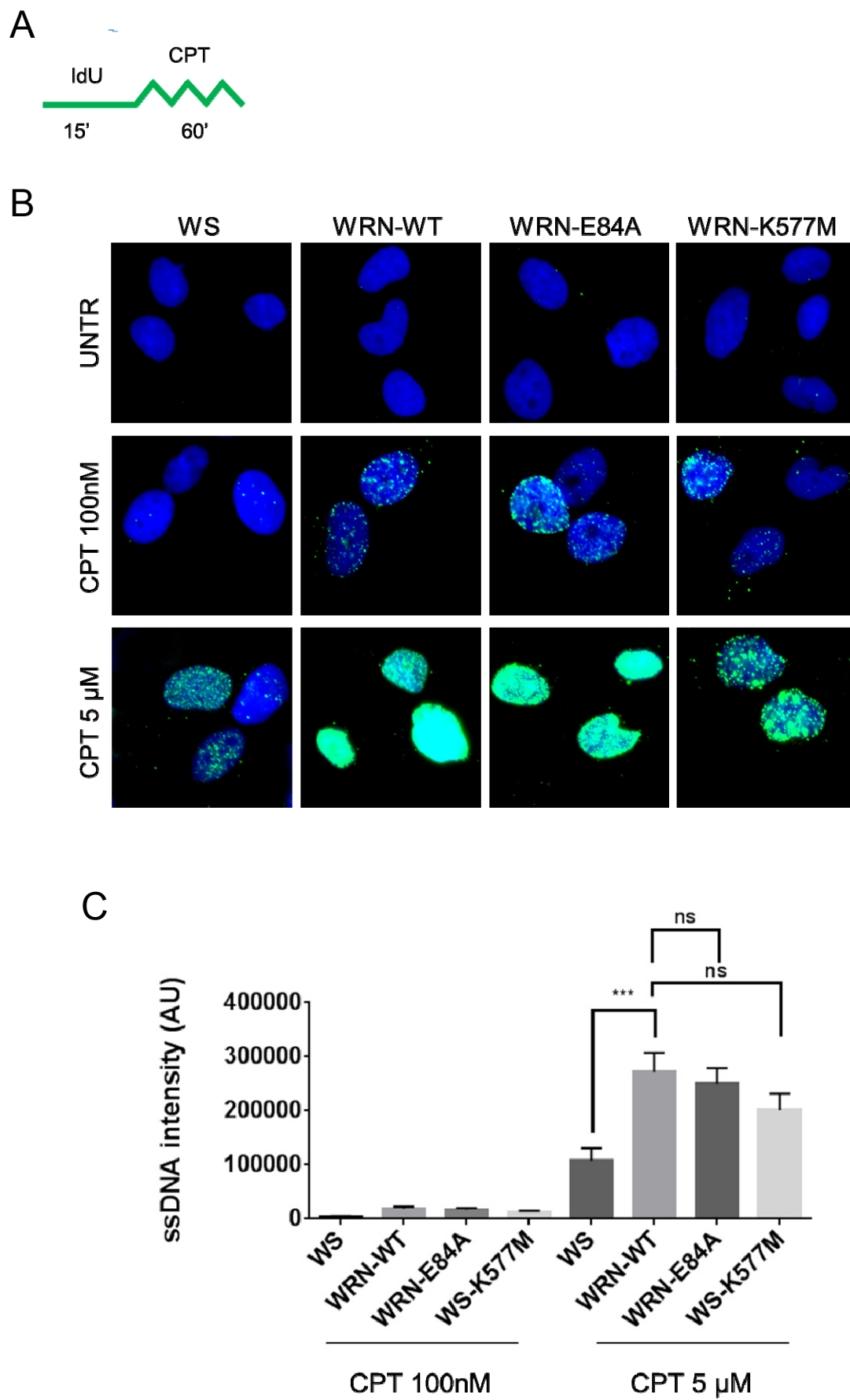

Figure S5

A

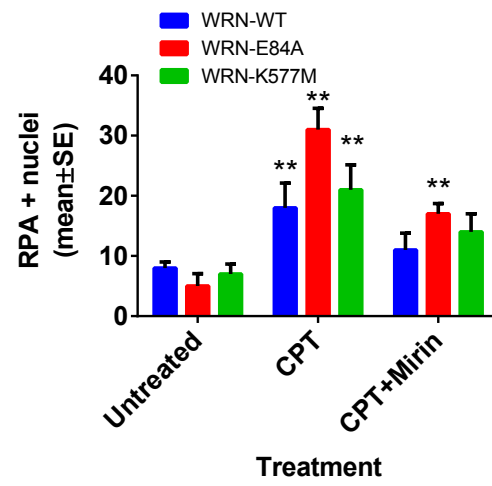

B

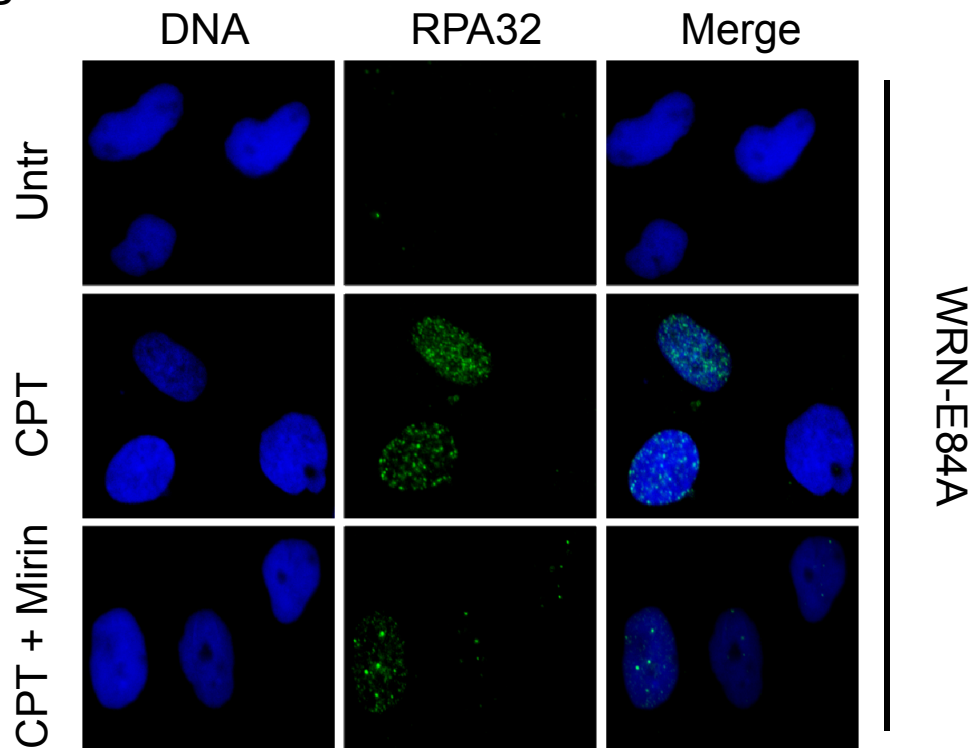

Figure S6

C

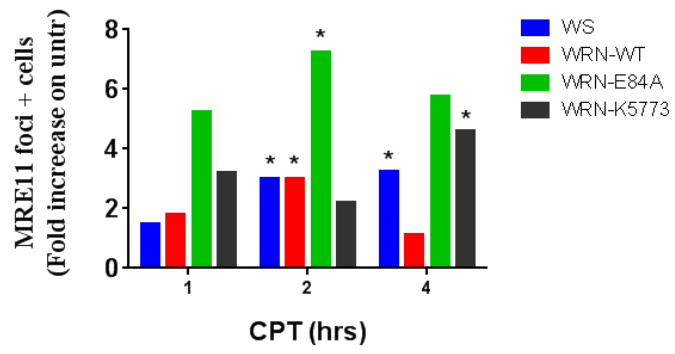

D

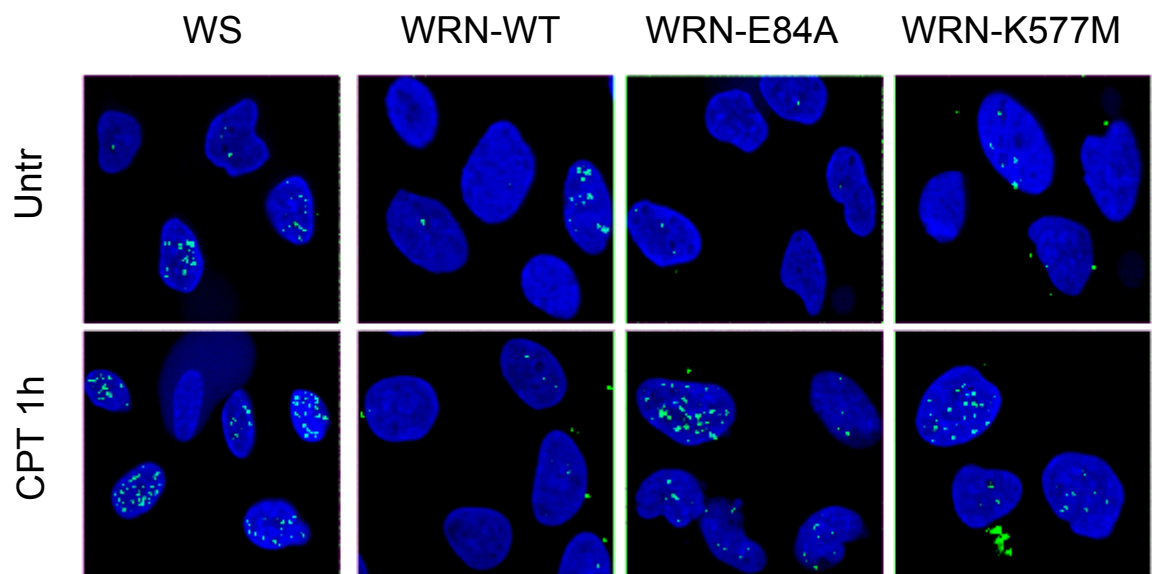

Figure S6 cont'd

A

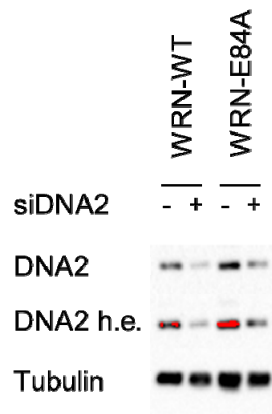

B

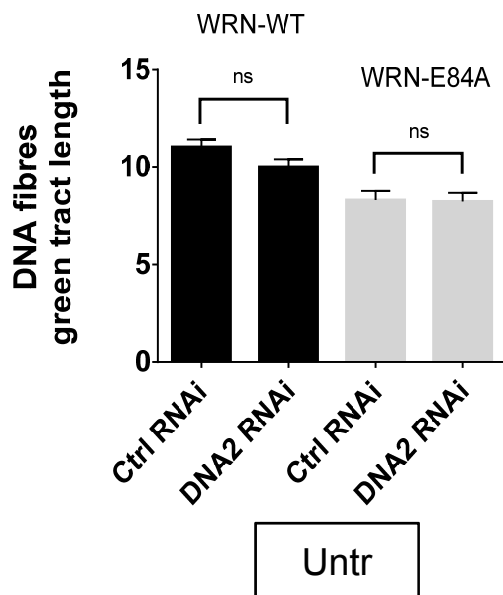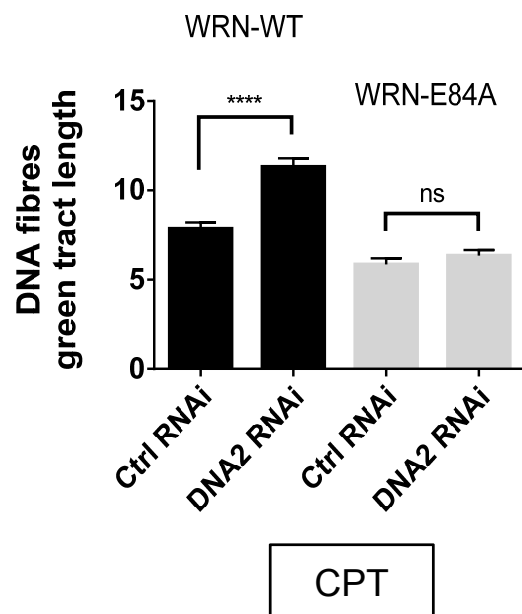

C

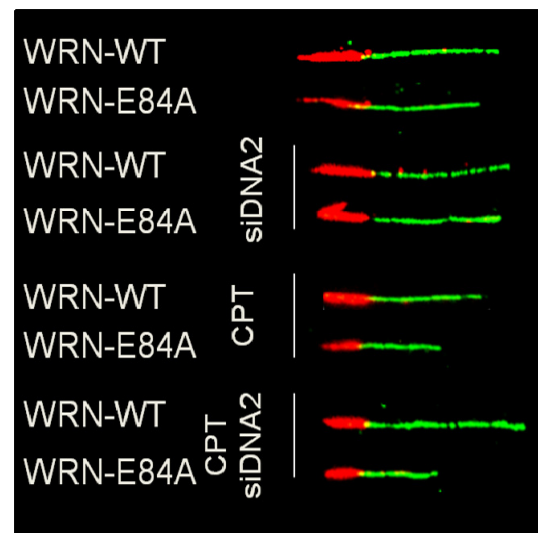

D

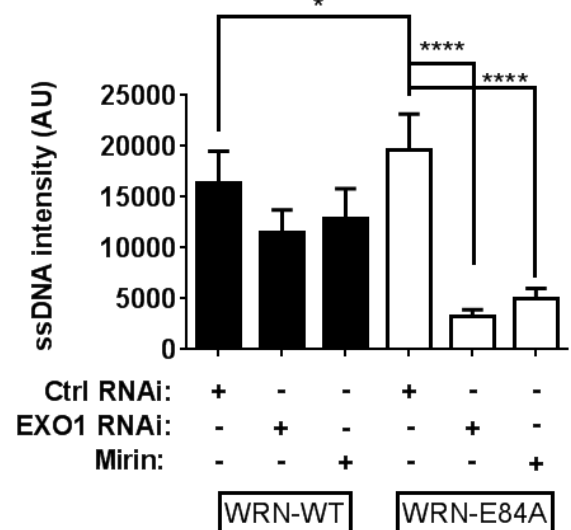

Figure S7

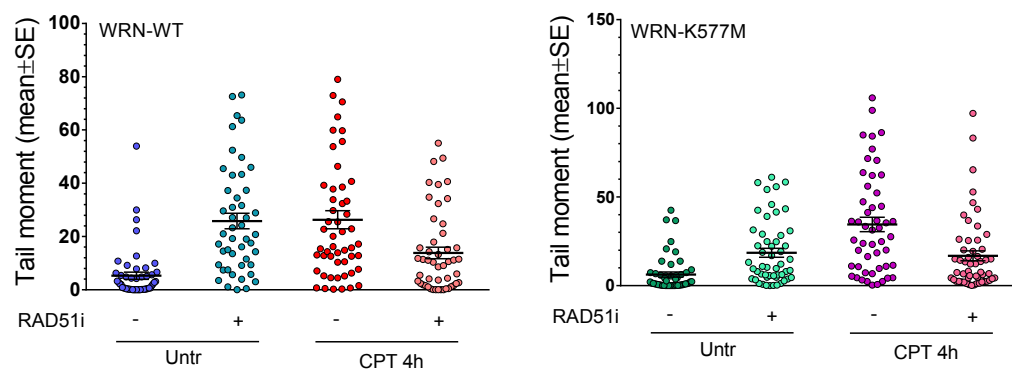

Figure S8

Supplement: SUPPLEMENTARY DATA [file supp_gkv836_nar-00551-v-2015-File014.pdf]
